# Sternal Osteomyelitis Caused by *Mycobacterium tuberculosis* in a Heart Transplant Patient

**DOI:** 10.1016/j.jaccas.2024.103088

**Published:** 2025-03-05

**Authors:** David A. Ocampo, Camilo A. Hernández, Fernan Mendoza, Carlos Orozco, William M. Rios

**Affiliations:** a Faculty of Medicine, El Bosque University, Bogotá, Colombia; b Department of Cardiology, Shaio Clinic Foundation, Bogotá, Colombia; c Department of Pathology, Shaio Clinic Foundation, Bogotá, Colombia; d Department of Cardiovascular Surgery, Shaio Clinic Foundation, Bogotá, Colombia

**Keywords:** cardiac transplant, chronic heart failure, *Mycobacterium tuberculosis*

## Abstract

*Mycobacterium tuberculosis* (MT) infection poses a significant risk in immunocompromised individuals, particularly in areas with high MT prevalence. We describe the case of a 43-year-old heart transplant recipient who developed chronic sternal wound secretion post-transplant surgery. Imaging revealed signs of inflammatory tissue suggestive of chronic osteomyelitis. Removal of the sternal wires was performed, followed by biopsy, which identified granulomatous inflammatory tissue and Langhans cells, that led to the diagnosis of MT infection later confirmed in culture results. The patient completed a 6-month tetraconjugate antituberculous therapy, resulting in successful wound healing and infection resolution. Atypical extrapulmonary MT manifestations require high clinical suspicion, especially in immunocompromised patients, to ensure timely diagnosis and treatment with favorable outcomes.

## Visual Abstract

**Figure:**
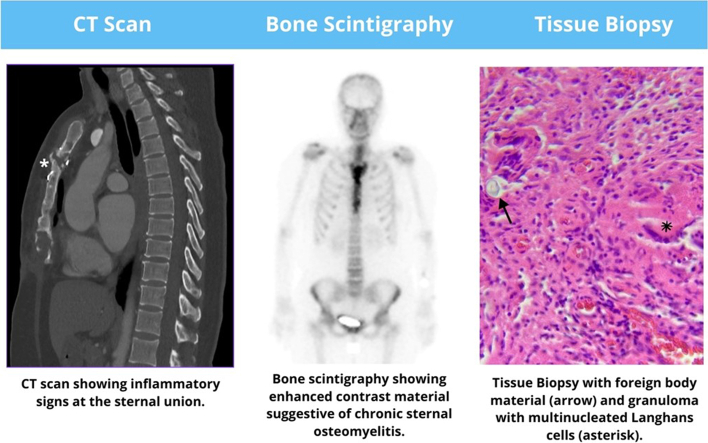

**Visual Summary:**
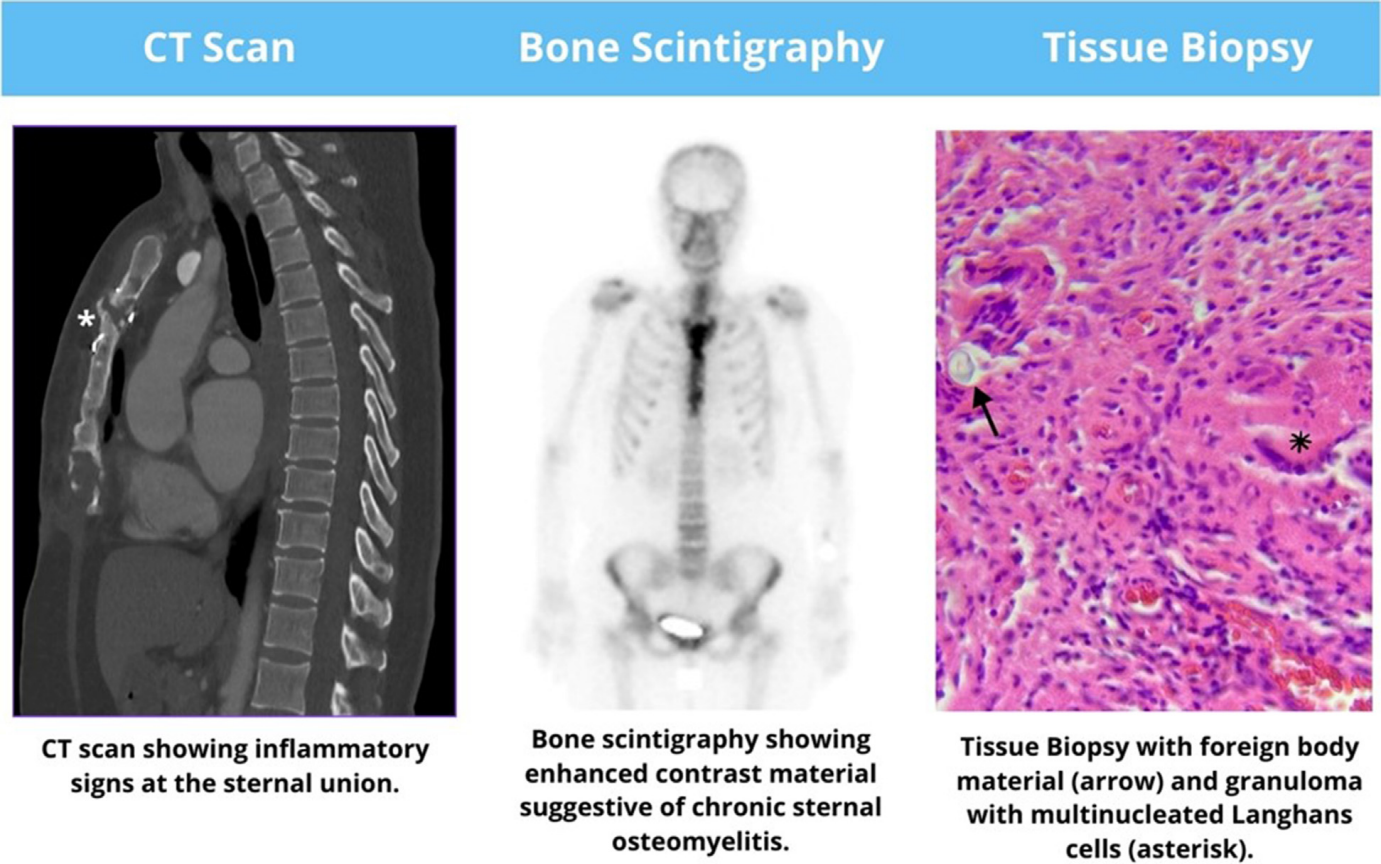
Sternal Osteomyelitis Caused by *Mycobacterium tuberculosis* After Heart Transplant

## Clinical Case Presentation

A 43-year-old female heart transplant recipient developed chronic sternal wound serous secretion and surrounding erythema 1 year after surgery. Except for the wound secretion, she was otherwise asymptomatic, without any other signs of localized or systemic inflammatory manifestations. On initial evaluation, her vital signs were stable, and the cardiovascular examination was normal, with no murmurs or abnormal findings.

**Take-Home Messages**

• A high clinical suspicion guided by multimodal diagnostic tools is necessary for prompt diagnosis and management of infectious complications after heart transplantation.
• Close follow-up after heart transplantation surgery is crucial to identify possible early and late complications, including infections that may occur even after years of transplantation as a result of immunosuppressive medications.
• When evaluating granulomas, MT infection is a critical condition that must be carefully considered in the differential diagnosis, especially in immunocompromised heart transplant recipients.

## Past Medical History

The patient had advanced heart failure secondary to Chagas cardiomyopathy, with a severely reduced ejection fraction despite optimal medical therapy. She underwent heart transplantation in 2019 without early complications. Her medical history was otherwise unremarkable, and her current medications included immunosuppressant agents prescribed for the heart transplant.

## Differential Diagnosis

The primary differential diagnosis for this chronic inflammatory reaction includes a foreign body reaction to the sternal wire. Infectious causes of granuloma formation are also leading considerations for chronic wounds in immunocompromised hosts. Granuloma formation, whether caused by infectious agents such as bacteria, tuberculous and nontuberculous mycobacteria, fungi, and parasites, or by noninfectious conditions, should be addressed in a systematic manner.^1^

## Investigations

**Figure 1:**
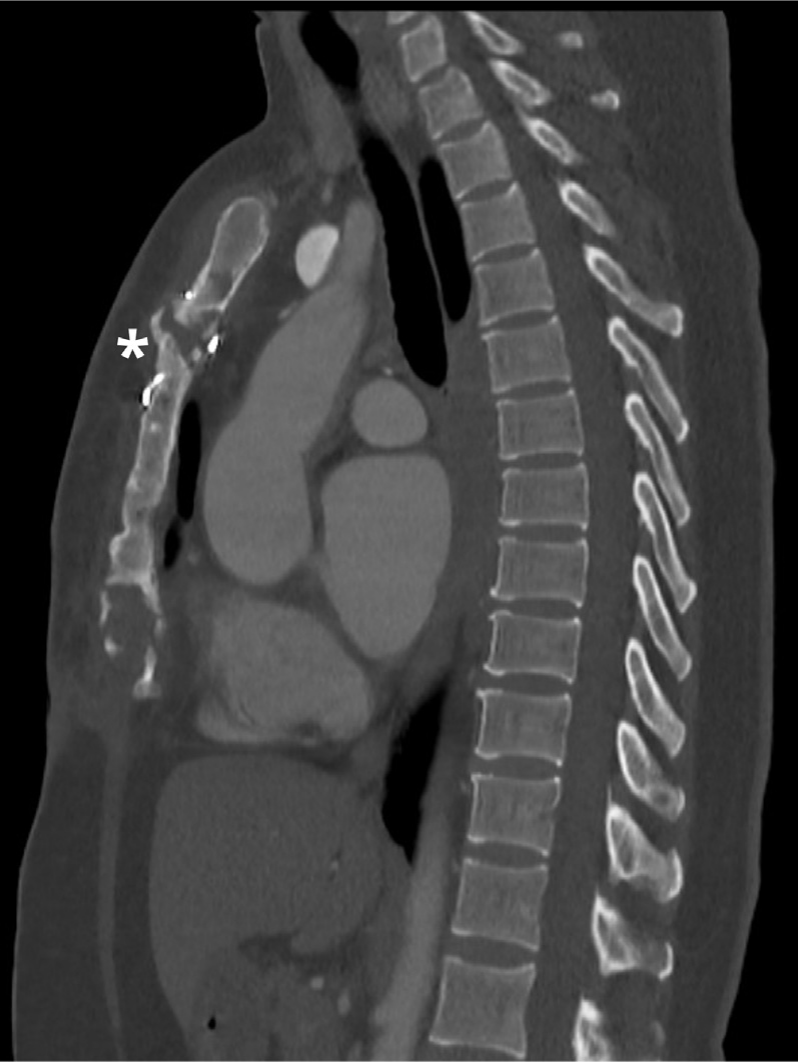
Chest Computed Tomography With Signs of Sternal Osteomyelitis Scan showing corticomedullary chronic sclerotic inflammatory signs at the body-manubrium sternal union (asterisk).

**Figure 2:**
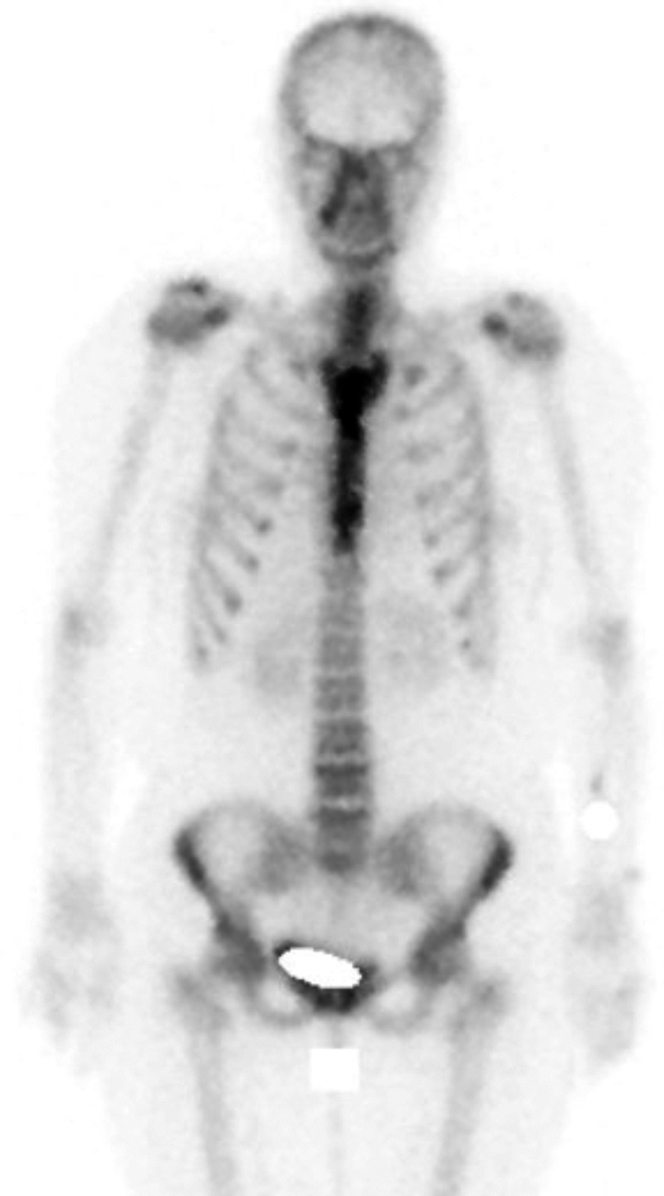
Bone Scintigraphy With Enhanced Contrast Material in the Sternum Enhanced contrast material in the sternum suggestive of chronic sternal osteomyelitis.

**Figure 3:**
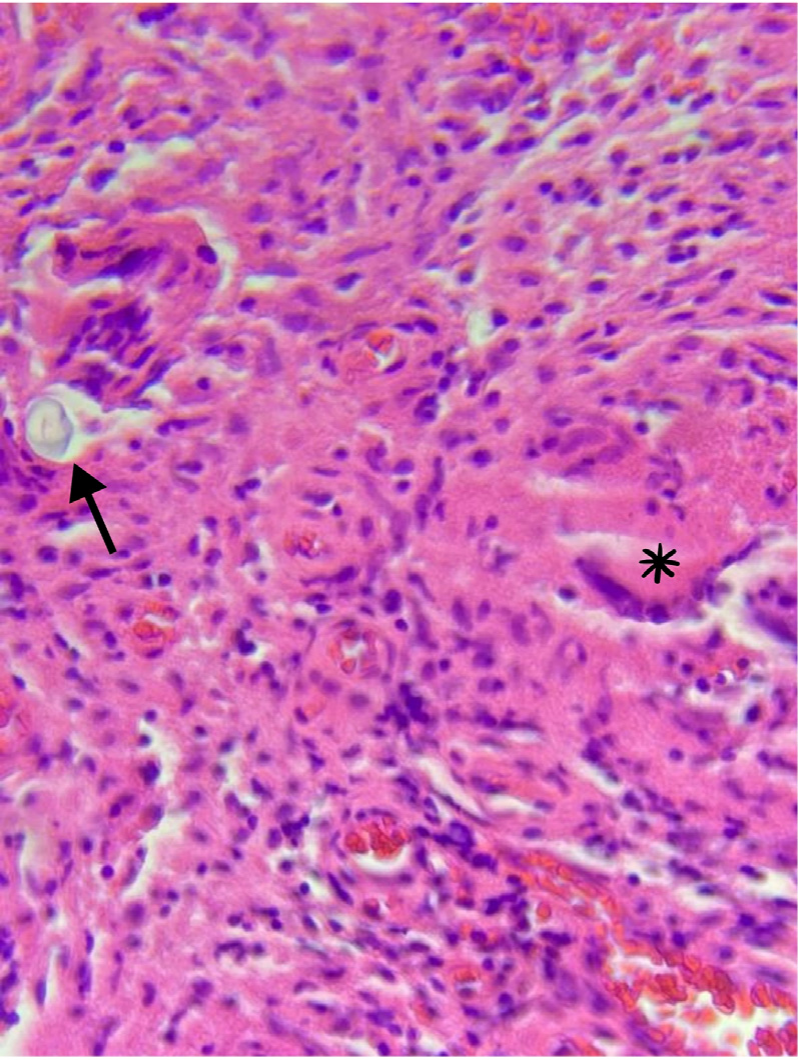
Bone Tissue Biopsy Showing Foreign Body Material and Granuloma Hematoxylin and eosin staining showing foreign body material (arrow) with a surrounding granuloma and multinucleated Langhans cells (asterisk).

Initial laboratory test results were unremarkable, showing no systemic inflammatory response. A computed tomography (CT) scan (Figure 1) revealed sternal bone sclerosis and surrounding inflammatory changes with soft tissue involvement. These findings initially suggested a granulomatous foreign body reaction to the sternal wire, prompting partial sternal cerclage and granuloma resection. A tissue biopsy showed foreign body material with surrounding granulomas, scar fibrosis, and granulation tissue. Despite regular wound clinic assessments, the patient continued to experience sternal discharge, which later became purulent during follow-up. Bone scintigraphy indicated enhanced contrast suggestive of chronic sternal osteomyelitis (Figure 2). A subsequent surgical procedure involved complete sternal cerclage removal. Although bacterial and fungal culture results were negative, another tissue biopsy revealed granulomas with multinucleated Langhans cells (Figure 3) that led to the diagnosis of MT infection, which was confirmed with culture results and molecular studies. Conventional rifampicin, isoniazid, pyrazinamide, and ethambutol (RHZE) treatment was started, guided by sensitivity culture tests.

## Management

The patient was started on a 6-month course of tetraconjugate antituberculous therapy, including RHZE. This treatment led to favorable wound healing and resolution of the infection.

## Discussion

MT infection remains a substantial public health concern, particularly affecting vulnerable persons such as immunocompromised individuals after solid organ transplantation, especially in regions with high MT prevalence.^2^ This infection can manifest as a pulmonary condition and less commonly as an extrapulmonary representation following heart transplantation, reported to be approximately 1.35 cases per 100 heart transplant-years. The mean interval time between transplantation and MT infection onset is 72 days post-transplantation,^3^ but this infection may occur even 1 year after the heart transplantation.

An impaired immune response in transplant recipients is mainly caused by immunosuppression medications that make individuals susceptible to tuberculosis and other infections, with an increase in complications and mortality.^4^ Tuberculosis can be acquired by donor-derived infection or by reactivation of latent tuberculosis, thus making screening prevention an important test before solid organ transplantation.

The diagnosis of an infectious complication caused by MT after heart transplantation presents a significant challenge because of the often atypical or insidious mild initial clinical presentation and limited inflammatory signs, which can be masked by post-transplantation immunosuppression therapy, essential for preventing allograft rejection. Immunosuppression dampens the immune host response, consequently blunting the clinical presentation, mimicking other conditions such as foreign body reactions or other complications, and delaying MT recognition and prompt diagnosis.^5^

Most MT infections after heart transplantation result from latent tuberculosis reactivation and can manifest even after years following transplantation. Early detection and management with a conventional antituberculosis regimen are crucial for favorable outcomes. Although drug-sensitive MT generally has a high cure rate with RHZE, solid organ transplant recipients face a significant increased mortality risk, with reported rates reaching up to 18%.^6^

A multimodal imaging approach may be necessary in difficult scenarios such as immunosuppressed patients for an accurate diagnosis. Although a CT scan is usually the first image obtained when suspecting deep tissue infections, other imaging tools may be necessary to identify bone infections, such as magnetic resonance or bone scintigraphy. In this clinical case, bone scintigraphy was a useful diagnostic tool that revealed and localized chronic sternal osteomyelitis. Given the broad range of differential diagnoses as causes of chronic inflammation in transplant recipients, a direct tissue biopsy may be necessary to identify a specific cause. Tissue biopsy of MT lesions displays a specific exudative inflammation that may vary depending on the time course: granulomatous lesions with a necrotic center and typical cells such as epithelioid macrophages and Langhans multinucleated giant cells.^7^ Once MT is identified, cultures and molecular testing systems are necessary for appropriate treatment given the increasing rate of resistant tuberculosis.

## Conclusions

This case highlights the atypical extrapulmonary presentation of MT infection as chronic sternal osteomyelitis in an immunocompromised patient 1 year after heart transplantation. Early recognition and treatment are essential for managing such cases, thus underscoring the need for a high index of suspicion and comprehensive diagnostic evaluation in immunocompromised individuals with chronic granulomas and deep tissue infections. The heightened susceptibility to opportunistic infections requires vigilant monitoring post-heart transplantation, to ensure long-term survival and minimize complications following immunosuppressive drug treatment.

## Funding Support and Author Disclosures

The authors have reported that they have no relationships relevant to the contents of this paper to disclose.
